# Oxidatively-induced DNA base damage and base excision repair abnormalities in siblings of individuals with bipolar disorder DNA damage and repair in bipolar disorder

**DOI:** 10.1038/s41398-024-02901-3

**Published:** 2024-05-24

**Authors:** Hidayet Ece Arat Çelik, Selda Yılmaz, İzel Cemre Akşahin, Burcu Kök Kendirlioğlu, Esma Çörekli, Nazlı Ecem Dal Bekar, Ömer Faruk Çelik, Neşe Yorguner, Bilge Targıtay Öztürk, Hüray İşlekel, Ayşegül Özerdem, Pınar Akan, Deniz Ceylan, Gamze Tuna

**Affiliations:** 1 Department of Psychiatry, School of Medicine, Maltepe University, Istanbul, Turkey; 2 Department of Molecular Medicine, Institute of Health Sciences, Dokuz Eylul University, Izmir, Turkey; 3 Graduate School of Health Sciences, Koc University, Istanbul, Turkey; 4 Research Center for Translational Medicine (KUTTAM), School of Medicine, Koc University, Istanbul, Turkey; 5 Chair of Proteomics and Bioanalytics, School of Life Sciences, Technical University of Munich, Munich, Germany; 6 Department of Medical Biochemistry, Sancaktepe Sehit Prof. Dr. Ilhan Varank Training and Research Hospital, Istanbul, Turkey; 7 Department of Psychiatry, School of Medicine, Marmara University, Istanbul, Turkey; 8 Department of Psychiatry, School of Medicine, Dokuz Eylul University, Izmir, Turkey; 9 Department of Medical Biochemistry, School of Medicine, Dokuz Eylul University, Izmir, Turkey; 10 Department of Psychiatry and Psychology, Mayo Clinic, Rochester, MN USA; 11 Department of Neuroscience, Institute of Health Sciences, Dokuz Eylul University, Izmir, Turkey; 12 BioIzmir - Izmir Health Technologies Development and Accelerator Research and Application Center, Dokuz Eylul University, Izmir, Turkey; 13 Department of Psychiatry, School of Medicine, Koc University, Istanbul, Turkey

**Keywords:** Molecular neuroscience, Diagnostic markers

## Abstract

Previous evidence suggests elevated levels of oxidatively-induced DNA damage, particularly 8-hydroxy-2’-deoxyguanosine (8-OH-dG), and abnormalities in the repair of 8-OH-dG by the base excision repair (BER) in bipolar disorder (BD). However, the genetic disposition of these abnormalities remains unknown. In this study, we aimed to investigate the levels of oxidatively-induced DNA damage and BER mechanisms in individuals with BD and their siblings, as compared to healthy controls (HCs). 46 individuals with BD, 41 siblings of individuals with BD, and 51 HCs were included in the study. Liquid chromatography-tandem mass spectrometry was employed to evaluate the levels of 8-OH-dG in urine, which were then normalized based on urine creatinine levels. The real-time-polymerase chain reaction was used to measure the expression levels of *8-oxoguanine DNA glycosylase 1 (OGG1), apurinic/apyrimidinic endonuclease 1 (APE1), poly ADP-ribose polymerase 1 (PARP1)*, and *DNA polymerase beta (POLβ)*. The levels of 8-OH-dG were found to be elevated in both individuals with BD and their siblings when compared to the HCs. The *OGG1* and *APE1* expressions were downregulated, while *POLβ* expressions were upregulated in both the patient and sibling groups compared to the HCs. Age, smoking status, and the number of depressive episodes had an impact on *APE1* expression levels in the patient group while body mass index, smoking status, and past psychiatric history had an impact on 8-OH-dG levels in siblings. Both individuals with BD and unaffected siblings presented similar abnormalities regarding oxidatively-induced DNA damage and BER, suggesting a link between abnormalities in DNA damage/BER mechanisms and familial susceptibility to BD. Our findings suggest that targeting the oxidatively-induced DNA damage and BER pathway could offer promising therapeutic strategies for reducing the risk of age-related diseases and comorbidities in individuals with a genetic predisposition to BD.

## Introduction

Bipolar disorder (BD) is a chronic mood disorder that often co-occurs with various medical illnesses and is associated with premature aging, resulting in a shortened life expectancy [1]. Mounting evidence suggests that individuals with BD have elevated levels of oxidatively-induced DNA damage [2–4]. DNA is a highly susceptible molecule to oxidative insults, and it is estimated that every single cell of the human body is exposed to up to a million DNA lesions [5, 6]. Nevertheless, these damages are repaired by the cellular DNA repair machinery, which includes the base excision repair (BER). However, an overload of oxidative insults or insufficient repair can lead to persistent DNA damage, genomic instability, and ultimately, premature aging and the development of various illnesses [6]. Therefore, oxidatively-induced DNA damage and abnormal DNA repair mechanisms have been suggested to play a crucial role in the shared pathophysiology among BD, increased cellular aging, and comorbidity [7–10].

The 8-hydroxy-2’-deoxyguanosine (8-OH-dG) is formed by the attack of the hydroxyl radical at the C8-position of guanine of dG followed by the one-electron oxidation of the OH-adduct radical of guanine [11, 12]. Since guanine is the most susceptible base to oxidation due to its low reduction potential, 8-OH-dG is the most widely used parameter to determine oxidatively-induced DNA damage [13, 14]. Alongside 8-hydroxyguanine (8-OH-Gua), various purine and pyrimidine base damages such as 8-hydroxyadenine (8-OH-Ade), 4,6-diamino-5-formamidopyrimidine (FapyAde), 2,6-diamino-4-hydroxy-5-formamidopyrimidine (FapyGua), cytosine glycol (Cyt gly), thymine glycol (Thy gly), 5-hydroxymethyluracil (5-OH-MeUra), and 5,6-dihydroxycytosine exist. In contrast to the numerous well-established oxidatively-induced DNA base lesions, earlier research in psychiatric disorders exclusively concentrated on 8-OH-dG. Higher levels of 8-OH-dG were shown in individuals with BD compared to healthy controls (HCs) [2–4]. While some studies found higher levels during manic and depressive episodes, but not during euthymia [15–17], others found increased levels during all phases, including euthymia [18, 19]. A meta-analysis suggested that the increase of the 8-OH-dG is more pronounced in the depressive state of BD [4].

There are various DNA repair mechanisms, each designed to address specific types of damage or lesions, including oxidatively induced DNA lesions such as 8-OH-dG. Abnormal DNA repair processes have been observed in various medical conditions, including cancer and BD [20–23]. BER is recognized as the primary mechanism for addressing oxidatively-induced DNA damage [12], and while the number of studies on BER in BD is limited, reported abnormalities suggest its involvement in the disorder [24].

The BER pathway initiates with the recognition and removal of the damaged base by specific enzymes called DNA glycosylases, forming an apurinic/apyrimidinic (abasic) site. Among these, 8-oxoguanine DNA glycosylase 1 (OGG1) is specialized for the excision of 8-hydroxyguanine lesions. While existing research on BER gene expression in individuals with BD has predominantly focused on OGG1, showing decreased expression levels [25–27], the roles of genes involved in subsequent steps after DNA glycosylases remain less explored in BD.

In the BER pathway, endonucleases take part after DNA glycosylases and cleave the phosphodiester bonds in this abasic region. Apurinic/apyrimidinic endonuclease 1 (APE1) is a multifunctional endonuclease that creates a single-strand break on DNA, and the relationship between APE1 polymorphism and different cancer types has been identified in previous research [20, 28]. In addition, a study found that the polymorphism of APE1 is associated with the risk and onset of depression in patients with recurrent depression [29]. Subsequently, Poly-ADP-ribose polymerase binds to the broken DNA ends, protecting them from further degradation. Finally, the gap in the site is filled by DNA polymerases, and a phosphodiester bond is formed by DNA ligases [30–33]. Downregulation of *DNA polymerase gamma (POLG)* gene expressions [26], and upregulation of *poly ADP-ribose polymerase 1 (PARP1)* gene expression [34] have been reported in mood disorders. DNA polymerase beta (POLβ) is the key polymerase enzyme of the BER mechanism responsible for the addition of nucleotides to apurinic/apyrimidinic ends. It participates in various processes such as maintaining the stability of the genome [35] and telomeres [36] in cells, meiosis [37], and the ligation of non-homologous ends [38, 39]. To date, there have been no studies on the role of *POLβ* gene expression levels in psychiatric disorders.

Given that BD is a highly heritable disease, studies conducted on high-risk individuals are of great importance for the early detection of the disease and the identification of biological risk factors. The number of studies investigating oxidatively-induced DNA damage in individuals at high risk for BD is limited so far. Coello and colleagues found that the levels of 8-OH-dG were higher in newly diagnosed BD patients and their first-degree relatives compared to HCs [40]. On the other hand, another study found no difference in the levels of 8-OH-dG between twins at risk for mood disorders and HCs [41].

Currently, there is a dearth of research examining both oxidatively-induced DNA damage and BER mechanisms in individuals with BD, as well as in those at risk for the disorder, such as siblings of affected individuals. Thus, this study aims to investigate the levels of 8-OH-dG and assess the expression levels of the BER pathway genes involved in repairing 8-OH-dG lesions in individuals with BD, siblings of individuals with BD, and healthy controls.

## Materials and methods

### Study design

This is an observational study with a cross-sectional design that includes case-control groups. The study included 46 individuals with BD who were followed up at the Maltepe University, Faculty of Medicine, Department of Psychiatry between 2021 and 2022, 41 siblings of individuals with BD, and 51 HCs. The siblings of patients who are currently being followed at the hospital were invited to participate in the study via phone contact. The HCs were selected from individuals who agreed to participate in the study by accepting the distributed leaflets around the hospital vicinity. The research has been approved by the Maltepe University Faculty of Medicine Clinical Research Ethics Committee. (Date: 07/24 /2019 Number: 2019/900/48).

### Participants

The individuals participating in the study were between the ages of 18–50. All participants underwent a SCID-5 interview in which the DSM-5 diagnostic criteria were examined. In addition, Health-Promoting Lifestyle Profile-II (HPLP-II) and World Health Organization Quality of Life-BREF (WHOQOL-Bref) were applied to the participants. While some studies found differences between affective episodes and euthymia in oxidatively-induced DNA damage markers [15, 17], we only included individuals with BD or siblings who were in remission for at least 4 months. The participants with HAMD-17 and YMRS scores below 7 were included in the study. Individuals who had a decompensated systemic medical condition, morbid obesity, diabetes mellitus, rheumatologic disease, active infection, or a serious neurological disease that could affect oxidative parameters, using antioxidant-containing treatments or supportive products that could affect oxidative parameters, had a serious abnormality in routine laboratory findings, had a lifelong intellectual disability or conditions affecting cognitive function (such as delirium, dementia, epilepsy, etc.), had a diagnosis of alcohol or substance abuse or addiction at any point of life, were in pregnancy, lactation period or were going through menopause were excluded from the study. Individuals who were diagnosed with schizophrenia, schizophreniform disorder, schizoaffective disorder, brief psychotic disorder, and psychotic disorder not otherwise specified after the SCID-5 interview in the sibling group were excluded from the study, because recent studies demonstrated a significant increase in oxidatively-induced DNA damage in psychotic disorders [3]. On the other hand, siblings who had a history of or currently have any psychiatric diagnosis have not been excluded from the study, as they may have a higher predisposition towards BD. In the healthy control group, individuals who had a history of or currently had any psychiatric diagnosis were excluded from the study. Urine and blood samples were collected from the participants after the diagnostic interview.

### Collection and storage of the samples

Participants’ first-morning urine samples and fasting blood samples (10 ml) between 08:00 and 09:00 am were collected. Daily RNA isolations were performed on the collected whole blood samples. The RNA was extracted from each whole blood sample using RNeasy Mini Kit (Qiagen Diagnostics GmbH, Germany) following the manufacturer’s instructions. The amount and purity of RNA samples were measured using a Nanodrop 2000 spectrophotometer (Thermo Scientific). After isolation, RNA was stored at −80 °C until it was converted to cDNA.

### 8-OH-dG quantification

To evaluate oxidatively-induced DNA damage, liquid chromatography-tandem mass spectrometry (LC-MS/MS) with stable-isotope dilution using multiple reaction monitoring (MRM) acquisition mode was performed according to the previously published protocol [42]. 15.6 µL of 8-OH-dG-^15^N_5_ (0.002 mM) internal standard is added to 1 mL of urine samples [43], which were then centrifuged at 1000 × *g* for 15 min. Subsequently, supernatants were filtered using nylon syringe filters. Filtered samples were loaded onto extraction cartridges and then washed with 2 mL of distilled water. 1 mL of 30% methanol was used for the elution of retained material. Eluted samples were dried in a vacuum concentrator (Thermo Scientific SpeedVac, Marietta, Ohio, USA) and then dissolved in 100 µL digestion buffer (1 mol/L sodium acetate, 10 mmol/L Tris-HCl, pH 7.5). Then, samples were hydrolyzed with alkaline phosphatase (22 units per sample) at 37 °C for 1 h. Samples were filtered using 3 kDa tubes by centrifugation at 5000 × *g* for 50 min. The studies were performed by HPLC (Shimadzu, Kyoto, Japan) coupled with a mass spectrometer equipped with a triple quadrupole ion trap (4000 QTRAP Applied Biosystems, CA, USA) in the positive ionization mode. Samples were separated by an LC column with 2.1 mm × 150 mm, 3.5 µm particle size (Zorbax SB-Aq column, Agilent Technologies, California, USA) and an attached C8 guard column (2.1 mm × 12.5 mm, 5 µm particle size). Mobile phases were water containing 0.1% formic acid (mobile phase A) and acetonitrile containing 0.1% formic acid (mobile phase B). Analysis by LC-MS/MS with MRM was performed using the mass/charge (*m/z*) transitions *m/z* 284 → *m/z* 168, and *m/z* 289 → *m/z* 173 for 8-OH-dG and 8-OH-dG-^15^N_5_, respectively. Urinary creatinine concentrations were used for the normalization of the results. The results were expressed in nmol 8-OH-dG/mmol creatinine.

### Base excision enzymes mRNA expression quantification using quantitative real Time-PCR

RNA samples were converted to cDNA using the RT2 First Strand Kit (Qiagen Diagnostics GmbH, Germany), and the quantitative Reverse Transcription PCR amplification was performed in triplicate by The LightCycler® 480 Instrument II (Roche) at Koc University, KUTTAM Laboratory. *OGG1, APE1, PARP1*, and *POLβ* expressions were measured by RT-qPCR according to the manufacturer’s protocol (LightCycler 480 SY Green I Master Handbook Version 13, Roche Diagnostics GmbH, Mannheim, Germany). The *GAPDH* and *β-Actin* were used as housekeeping genes. The primers for *OGG1, PARP1, APE1*, *POLβ, GAPDH*, and *β-Actin* were obtained from the manufacturer (SentebioLab, Turkey) (Table 1). The outputs showing cycling reports and melting curves were obtained using the LightCycler® 480 Software Version 1.5.0.39. Any Ct value of more than 35 was considered negative melting curve and was analyzed to confirm the specificities of the amplicons for RT-qPCR amplification.

**Table 1:** Gene sequences of BER enzymes.

Gene	Gene sequences	
	F (5’-3’)	R (3’-5’)
OGG1	GGCTCAACTGTATCACCACTGG	GGCGATGTTGTTGTTGGAGGAAC
APE1	CTGCTCTTGGAATGTGGATGGG	TCCAGGCAGCTCCTGAAGTTCA
PARP1	CCAAGCCAGTTCAGGACCTCAT	GGATCTGCCTTTTGCTCAGCTTC
POLβ	TGCAGAGTCCAGTGGTGACATG	ATGAACCTTTTGTAACTGCTCCAC
GAPDH	CCCTTCATTGACCTCAACTACA	ATGACAAGCTTCCCGTTCTC
ACTB	CCCAGATCATGTTTGAGACCTT	CCAGAGGCGTACAGGGAT

Each set of samples underwent normalization using two housekeeping genes, *GAPDH* and *β-Actin*. The normalization was performed using the formula ΔCt = Ct _*OGG1*_ − [(Ct _*GAPDH*_ + Ct _*ß-Actin*_)/2]. To determine the relative changes in mRNA expression levels of the base excision enzyme genes, the term 2^−ΔΔCt^ was employed, where ΔΔCt = ΔCt _patient_ − ΔCt _the mean value of the healthy control group_, and the fold changes were compared between groups. The experiments adhered to the guidelines provided by Minimum Information about Quantitative Real-Time PCR Experiments (MIQE) [44].

### Statistical analyses

IBM SPSS Statistics 29.0 (Chicago IL, USA) for Windows was used for the statistical analysis. Categorical variables were analyzed with the Chi-square test. Continuous variables were checked for Gaussian distribution using quantile-quantile plots, distribution of data in histograms, skewness values (−1 to +1), kurtosis values (−2 to +2), and confirmed by the Kolmogorov–Smirnov Test. Medians and interquartile ranges were used in the figures. Both mean and SD and median and minimum–maximum values were presented in the tables when normal distribution was not achieved. The level of significance was taken as 0.05 for all tests.

Univariate Analysis of Variance (ANOVA) and Chi-square tests were applied to compare demographical and clinical variables between study groups. Levels of 8-OH-dG/creatinine and expression levels of *OGG1, APE1, PARP1, POLβ* were compared among study groups using the Quade Nonparametric Univariate Analyses of Covariance (ANCOVA) models which include age, sex, body mass index (BMI), smoking status, and alcohol consumption as covariates.

Spearman correlation analyses were applied in patient and sibling groups separately to evaluate correlations among continuous clinical variables (e.g., number of previous manic, hypomanic, and depressive episodes, number of episodes with mixed features, number of psychotic episodes, age of onset, duration of illness, duration of remission, number of suicide attempt, number of hospitalization, scale scores [HAMD-17, YMRS, HPLP-II total scores, WHOQOL-Bref total scores], etc.) and dependent variables (i.e., 8-OH-dG/creatinine levels, and *OGG1, APE1, PARP1, POLβ* gene expression levels). Point-biserial correlation analyses were applied to evaluate correlations among categorical clinical variables (e.g., current/past psychiatric history, medication type) and dependent variables (i.e., 8-OH-dG/creatinine levels, and *OGG1, APE1, PARP1, POLβ* gene expression levels).

Linear regression models were applied in patient and sibling groups separately, to identify the effect of sociodemographic variables (i.e., age, sex, BMI, smoking status, alcohol consumption, and HPLP-II total scores), and clinical variables that correlate with the main findings, current and past history of psychiatric illness, and medication use (i.e., mood stabilizers, antipsychotics, and antidepressants) on main findings.

## Results

### Demographics

Comparison of the demographic and clinical characteristics between study groups is presented in Table 2. While there was no significant difference in age, sex, and years of education between groups, a significant difference was found in terms of employment status (*p* < 0.001), marital status (*p* = 0.035), BMI (*p* = 0.007) and alcohol consumption (*p* = 0.033).

**Table 2:** Comparison of the demographic and clinical characteristics between study groups.

	BD (*n* = 46) Median (min-max) Mean ± SD	Sibling *n* = 41) Median (min-max) Mean ± SD	HC (*n* = 51) Median (min-max) Mean ± SD	Test statistics F/χ2	*p*
Age	35.33 ± 7.75	36.46 ± 7.86	34.25 ± 7.22	0.971	0.384
Female (*n*, %)	31 (67.4)	25 (61.0)	27 (52.9)	2.353	0.346
Years of education	15 (8–12) 14.54 ± 2.47	15 (8–23) 14.12 ± 4.01	15 (7–22) 14.61 ± 2.70	0.307	0.727
Employed (*n*, %)	31 (67.4)	35 (85.4)	50 (98)	18.385	**<0.001**
Married (*n*, %)	19 (41.3)	28 (68.3)	30 (58.8)	6.131	**0.035**
BMI	27.01 ± 4.61	24.79 ± 3.45	24.83 ± 3.11	6.278	**0.007** **BD** **>** **Sibling** ***p*** = **0.007** **BD** **>** **HC** ***p*** = **0.005**
Smoker (*n*, %)	27 (58.7)	17 (41.5)	28 (54.9)	3.064	0.244
Using alcohol (*n*, %)	19 (41.3)	25 (61.0)	34 (66.7)	7.397	**0.033**
HPLP-II					
*Health responsibility*	21 (13–30) 21.25 ± 4.16	21 (11–27) 20.54 ± 4.13	21 (14–30) 21.12 ± 3.88	0.365	0.695
*Physical activity*	15 (8–27 15.25 ± 4.64	17 (9–30) 17.36 ± 5.04	18 (8–30) 17.76 ± 5.49	3.295	**0.040** **BD** **<** **HC** ***p*** **=** **0.016**
*Nutrition*	19 (12–29) 19.27 ± 3.83	20 (13–29 20.66 ± 3.47	20 (13–29) 19.99 ± 3.50	1.619	0.202
*Spiritual growth*	26 (11–34 25.20 ± 4.65	26 (18–35) 26.22 ± 4.13	28 (18–34 27.53 ± 3.68	3.857	**0.023** **BD** **<** **HC** ***p*** = **0.007**
*Interpersonal relations*	25.08 ± 4.29	25.89 ± 3.98	27.04 ± 3.22	3.223	**0.043** **BD** **<** **HC** ***p*** = **0.013**
*Stress management*	20.02 ± 3.50	10.90 ± 3.80	19.94 ± 3.31	0.013	0.987
*Total score*	126.06 ± 16.22	130.57 ± 16.59	133.38 ± 16.45	2.426	0.092
WHOQOL-Bref					
*Physical*	26 (19–33 26.29 ± 3.49	29 (16–35) 27.86 ± 4.13	30 (22–35) 29.91 ± 3.21	12.361	**<0.001** **BD** **<** **HC** ***p*** **<** **0.001** **BD** **<** **Sibling** ***p*** **=** **0.045** **Sibling** **<** **HC** ***p*** **=** **0.007**
*Psychological*	22 (13–28) 21.24 ± 3.35	22 (14–28 21.90 ± 3.02	23 (15–30) 23.61 ± 2.85	7.702	**<0.001** **BD** **<** **HC** ***p*** **<** **0.001** **Sibling** **<** **HC** ***p*** **=** **0.009**
*Social*	10 (5–14) 10.37 ± 2.10	12 (8–14) 11.40 ± 1.80	12 (8–15) 11.82 ± 1.73	7.467	**< 0.001** **BD** **<** **HC** ***p*** **<** **0.001** **BD** **<** **Sibling** ***p*** **=** **0.012**
*Environment*	30 (22–40) 29.83 ± 4.08	31 (23–37) 30.72 ± 3.81	31 (23–39) 31.00 ± 3.33	1.261	0.287
*Total score*	94.77 ± 11.65	99.01 ± 11.63	103.91 ± 10.25	8.154	**<0.001** **BD** **<** **HC** ***p*** **<** **0.001** **Sibling** **<** **HC** ***p*** **=** **0.038**
Additional current psychiatric condition^a^ (*n*, %)	7 (15.2)	5 (12.2)	-	-	**-**
Past psychiatric condition^b^ (*n*, %)	1 (2.2)	13 (31.7)	-	-	**-**
Number of suicide attempts	0 (0–3) 0.28 ± 0.66	0 (0–1) 0.07 ± 0.26	-	-	-
Number of manic episodes	2 (1–10) 2.74 ± 1.96	-	-	-	-
Number of hypomanic episodes	0 (0–9) 0.80 ± 1.72	-	-	-	-
Number of depressive episodes	1 (0–10) 1.96 ± 1.91	-	-	-	**-**
Number of episodes with mixed features	0 (0–5) 0.43 ± 0.93	-	-	-	**-**
Number of psychotic episodes	2 (0–10) 2.13 ± 2.20	-	-	-	**-**
Total number of episodes	5 (2–20) 5.91 ± 3.60	-	-	-	**-**
Age of onset	22 (13–40) 22.72 ± 5.92	-	-	-	**-**
Duration of illness (months)	120 (24–396) 149.48 ± 94.82	-	-	-	**-**
Duration of remission (months)	12 (4–96) 22.22 ± 23.08	-	-	-	-
Number of hospitalizations	1 (0–8) 1.71 ± 1.73	-	-	-	-
HAMD-17	0 (0–6) 0.98 ± 1.61	0 (0–0) 0.51 ± 1.07	-	-	-
YMRS	0 (0–4) 0.59 ± 1.13	0 (0–2) 0.05 ± 0.32	-	-	-

*BD* bipolar disorder, *HC* Healthy control, *BMI* Body mass index, *HPLP II* Health-Promoting Lifestyle Profile-II, *WHOQOL-Bref* World Health Organization Quality of Life-BREF, *HAMD-17* Hamilton Depression Rating Scale-17, *YMRS* Young Mania Rating Scale.

^a^Additional current psychiatric conditions for BD patients: anxiety disorders (*n* = 3), obsessive-compulsive disorder (OCD, *n* = 1), attention-deficiency/hyperactivity disorder (ADHD, *n* = 2), eating disorder (*n* = 1); for siblings: anxiety disorders (*n* = 3), comorbid anxiety and ADHD (*n* = 2).

^b^Past psychiatric condition for BD patients: ADHD (*n* = 1), for siblings: major depressive disorder (MDD, *n* = 10), anxiety disorders (*n* = 2), comorbid anxiety and ADHD (*n* = 1).

All significant results have been highlighted in bold.

### Urine 8-OH-dG/creatinine levels

**Fig. 1:**
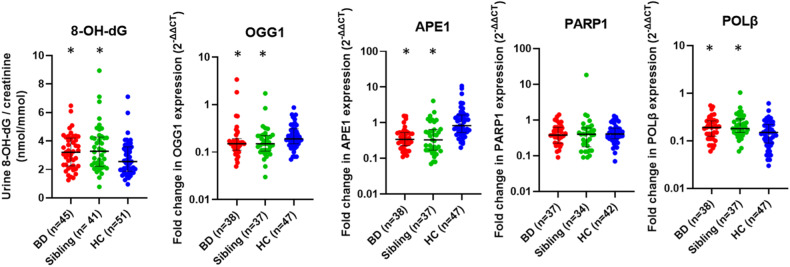
Comparison of 8-OH-dG/creatinine levels and BER pathway enzymes gene expression levels among study groups. Levels of 8-OHdG were significantly higher in individuals with bipolar disorder and siblings of individuals with bipolar disorder compared to healthy controls. Expression levels of *OGG1* and *APE1* genes were significantly lower, and expression levels of *POLβ* gene were significantly higher in individuals with bipolar disorder and siblings of individuals with bipolar disorder compared to healthy controls.

**Fig. 2:**
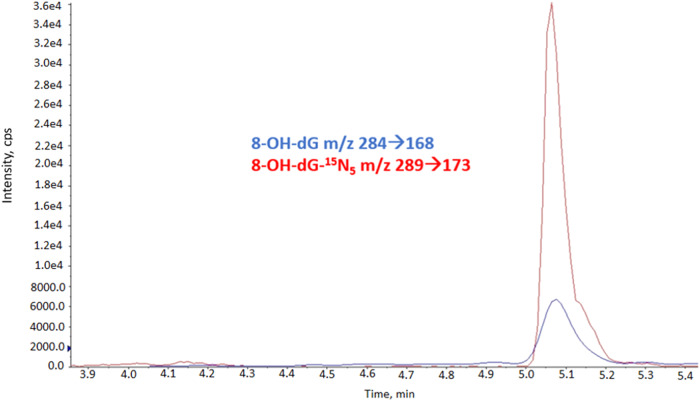
Ion–current profiles of the *m*/*z* 284→168 (8-OH-dG), *m*/*z* 289→173 (8-OH-dG-^15^N_5_) mass transitions. Ion–current profiles for 8-OH-dG at *m*/*z* 284→168 (*m*/*z* precursor ion → product ion) and for 8-OH-dG-^1^^5^N_5_ at *m*/*z* 289→173 (*m*/*z* precursor ion → product ion) were recorded during the LC-MS/MS analysis of a urine sample. The peak corresponding to 8-OH-dG in the figure originates from the analyte in the sample, and the peak for 8-OH-dG-^15^N_5_ comes from the stable isotope-labeled internal standard added to the sample. Quantification was performed using the ratio of the integrated peak areas of these two peaks.

The median urine 8-OH-dG levels were 3.20 (1.25–6.47) nmol/mmol creatinine in individuals with BD, 3.28 (0.78–8.94) nmol/mmol creatinine in siblings, and 2.55 (0.96–7.10) nmol/mmol creatinine in HCs (Table 3, Fig. 1). The levels of 8-OH-dG of individuals with BD and siblings were found to be significantly higher than the HCs (*F* = 4.520, *t* = 2.249, *p* = 0.026; *t* = 2.803, *p* = 0.006). There was no significant difference between individuals with BD and siblings according to 8-OH-dG levels. Figure 2 illustrates representative ion-current profiles of the m/z 284 → 168 (8-OH-dG), and m/z 289 → 173 (8-OH-dG-^15^N_5_) mass transitions, which were recorded during the LC-MS/MS analysis of urine samples.

**Table 3:** Comparison of 8-OH-dG/creatinine levels and BER pathway enzymes gene expression levels among study groups.

	BD Median (min-max) Mean ± SD	Sibling Median (min-max) Mean ± SD	HC Median (min-max) Mean ± SD	*F*	*p*
8-OH-dG/creatinine (nmol/mmol)	3.20 (1.25–6.47) 3.25 ± 1.22	3.28 (0.78–8.94) 3.51 ± 1.64	2.55 (0.96–7.10) 2.78 ± 1.19	4.520	**0.013** **BD** > **HC** ***p*** = **0.026** **Sibling** > **HC** ***p*** = **0.006**
Fold change in OGG1 mRNA expression (2^-ΔΔCt^)	0.15 (0.05–3.38) 0.30 ± 0.59	0.15 (0.03–1.72) 0.22 ± 0.29	0.19 (0.07–0.87) 0.25 ± 0.17	3.730	**0.027** **BD** < **HC** ***p*** = **0.020** **Sibling** < **HC** ***p*** = **0.025**
Fold change in APE1 mRNA expression (2^-ΔΔCt^)	0.34 (0.11–1.55) 0.45 ± 0.36	0.33 (0.07–4.08) 0.60 ± 0.75	0.83 (0.20–10.74) 1.60 ± 2.11	19.928	**<0.001** **BD** < **HC** ***p*** < **0.001** **Sibling** < **HC** ***p*** < **0.001**
Fold change in PARP1 mRNA expression (2^-ΔΔCt^)	0.38 (0.09–1.42) 0.49 ± 0.31	0.40 (0.09–18.32) 0.96 ± 3.08	0.40 (0.07–1.30) 0.50 ± 0.30	0.301	0.741
Fold change in POLβ mRNA expression (2^-ΔΔCt^)	0.19 (0.06–0.55) 0.21 ± 0.12	0.18 (0.06–1.03) 0.24 ± 0.17	0.15 (0.03–0.61) 0.16 ± 0.11	5.642	**0.005** **BD** > **HC** ***p*** = **0.014** **Sibling** > **HC** ***p*** = **0.002**

All significant results have been highlighted in bold.

### Base excision gene expression levels

The median *OGG1* mRNA expression levels of the patient group (0.15 [0.05–3.38]) and sibling group (0.15 [0.03–1.72]) were significantly lower than the control group (0.19 [0.07–0.87]) (*F* = 3.730, *t* = −2.361, *p* = 0.020; *t* = −2.278, *p* = 0.025). There was no significant difference between individuals with BD and siblings according to OGG1 mRNA expression levels.

The median *APE1* mRNA expression levels of the individuals with BD (0.34 [0.11–1.55]), and siblings (0.33 [0.07–4.08]) were significantly lower than the HCs (0.83 [0.20–10.74]) (*F* = 19.928, *t* = –5.597, *p* < 0.001; *t* = –5.101, *p* < 0.001). Individuals with BD and siblings showed no difference according to APE1 mRNA expression levels.

The median *PARP1* expression levels of the individuals with BD (0.38 [0.09–1.42]), siblings (0.40 [0.09–18.32]), and healthy controls (0.40 [0.07–1.30]) did not show a significant difference (*F* = 0.301, *p* = 0.741).

The median *POLβ* mRNA expression levels of individuals with BD (0.19 [0.06–0.55]) and siblings (0.18 [0.06–1.03]) were significantly higher than the HCs (0.15 [0.03–0.61]) (*F* = 5.642, *t* = 2.490, *p* = 0.014; *t* = 3.126, *p* = 0.002). Individuals with BD and siblings showed no difference according to *POLβ* mRNA expression levels (Table 3, Fig. 1).

### Effect of clinical variables on urine 8-OH-dG/creatinine and base excision gene expression levels

In the patient group, there was no correlation between demographic and clinical variables and 8-OH-dG/creatinine levels. There was a negative correlation between *OGG1* mRNA expression levels and the number of suicide attempts (*r* = −0.341, *p* = 0.036), HAMD-17 scores (*r* = −0.410, *p* = 0.013), and WHOQOL-Bref total scores (*r* = −0.376, *p* = 0.020). *APE1* mRNA expression levels showed positive correlations with the number of depressive episodes (*r* = 0.420, *p* = 0.009), and negative correlations with the number of hospitalizations (*r* = −0.330, *p* = 0.043) and WHOQOL-Bref total scores (*r* = −0.377, *p* = 0.020). *PARP1* mRNA expression levels showed a positive correlation with the use of antidepressants (*ρ* = 0.419, *p* = 0.010), and *POLβ* mRNA expression levels showed no correlation with the demographic and clinical variables. In the patient group, linear regression models, which included sociodemographic variables, and clinical variables that correlated with the main findings (i.e., number of depressive episodes, number of suicide attempts, number of hospitalizations, HAMD-17 scores, antidepressant use), revealed that those parameters did not have a significant impact on 8-OH-dG levels (R^2^ = 0.091, *F* = 0.635, *p* = 0.702). Age, smoking status, and the number of depressive episodes had an impact on *APE1* mRNA expression levels (R^2^ = 0.610, *F* = 5.659, *p* < 0.001; *B* = −0.016, *t* = −2.815, *p* = 0.009; *B* = 0.238, *t* = 2.653, *p* = 0.013; *B* = 0.124, *t* = 5.122, *p* < 0.001). Clinical parameters did not have an impact on *OGG1, PARP1* or *POLβ* mRNA expression levels (R^2^ = 0.314, *F* = 1.548, *p* = 0.187; R^2^ = 0.256, *F* = 1.422, *p* = 0.235; R^2^ = 0.258, *F* = 1.795, *p* = 0.133).

In siblings, there was a positive correlation between 8-OH-dG levels and past psychiatric history (*ρ* = 0.315, *p* = 0.045), and there was no relationship between *OGG1, APE1, POLβ, PARP1* expression levels, and clinical variables. In sibling group, linear regression models, which included sociodemographic variables, and clinical variables that correlated with the main findings (i.e., past psychiatric history), revealed that BMI, smoking status, and past psychiatric history had an impact on 8-OH-dG levels (R^2^ = 0.395, *F* = 3.073, *p* = 0.013; *B* = −0.174, *t* = −2.343, *p* = 0.025; *B* = 1.015, *t* = 2.129, *p* = 0.041; *B* = 1.338, *t* = 2.666, *p* = 0.012). None of the clinical variables had an impact on *OGG1, APE1, PARP1, POLβ* mRNA expression levels (R^2^ = 0.176, *F* = 1.065, *p* = 0.405; R^2^ = 0.180, *F* = 1.100, *p* = 0.385; R^2^ = 0.091, *F* = 0.453, *p* = 0.836; R^2^ = 0.213, *F* = 1.357, *p* = 0.264).

## Discussion

This study is the first to compare both oxidatively-induced DNA damage and BER expression levels among individuals with BD, siblings of individuals with BD, and HCs. Our findings indicate higher levels of 8-OH-dG, downregulated *OGG1*, and *APE1* expressions, and upregulated *POLβ* expressions in both the patient and sibling groups compared to HCs, with no significant differences observed between individuals with BD and their siblings.

Our findings present increased levels of 8-OH-dG in euthymic individuals with BD compared to HCs, which is supported by various studies in the literature [18, 19, 40]. Additionally, our results are consistent with a large-scale study that reported higher levels of 8-OH-dG in at-risk relatives of individuals with BD compared to HCs [40]. The elevated levels of 8-OH-dG in at-risk individuals support the role of oxidatively-induced DNA damage in BD pathogenesis. Additionally, our findings suggest that past psychiatric history has an impact on 8-OH-dG levels in siblings, most of whom had previous depressions. Increasing data suggest that there is a significant elevation in 8-OH-dG levels in depression [4, 45]. However, follow-up studies suggest that the increase in acute depression is reversible and returns to normal values after the resolution of depressive symptoms [27, 34, 46]. It should be noted that the siblings with previous episodes were euthymic at the time of inclusion in our study. Siblings with previous episodes may be more likely to share a genetic predisposition with individuals with BD, who present high levels of 8-OH-dG despite being euthymic. In addition, despite the widespread recognition of high DNA damage in obesity [47], we found that BMI has a negative impact on 8-OH-dG levels in siblings of individuals with BD. However, it is important to note that our study sample only included a small subset of siblings with obesity, and none of them had morbid obesity, which limits the generalizability of this finding.

Our finding showing a down-regulation in *OGG1* gene expression in BD is in concordance with two studies presenting consistent findings of decreased *OGG1* gene expression levels during euthymia in BD [25, 26]. In contrast, a study reported decreased *OGG1* gene expression in acute unipolar or bipolar depression compared to HCs, with a significant increase after remission [27]. However, the authors speculated that the increase in *OGG1* gene expression after remission might be temporary and may return to decreased levels over time. Our findings also present, a down-regulation of *APE1* gene expression and upregulation of *POLβ gene* expression in BD and sibling groups, while *PARP1* gene expression was comparable among study groups. As far as we know, *APE1* and *POLβ gene* expressions have not been investigated in peripheral samples of individuals with BD.

Our data revealed a significant association between *APE1* levels and the number of previous depressive episodes. Our findings also showed that *APE1* gene expression levels in patients with BD may be influenced by age, as oxidative stress has been shown to be closely related to aging and deficiencies in repair enzymes. Additionally, smoking had a positive effect on *APE1* gene expression levels in the patient group and 8-OH-dG levels in the sibling group, as smoking is associated with higher levels of 8-OH-dG. It is possible that increased expression of *APE1* levels occurs in response to increased levels of 8-OH-dG, serving as an adaptive mechanism for repair in individuals with BD. In our study, the finding of elevated *POLβ* gene expression levels in patient and sibling groups compared to HCs may indicate the significant involvement of this enzyme in the physiopathology of BD.

On the other hand, our study found comparable levels of *PARP1* gene expression in individuals with BD and their siblings to those in HCs. While previous research has not investigated *PARP1* levels in BD specifically, our negative result may be due to studying a group of individuals who were euthymic at the time of inclusion. PARP1 gene polymorphism has been found to be associated with depression [29], and increased *PARP1* gene expression levels have been observed in individuals with depression [34]. Inhibition of *PARP1* has shown antidepressant effects in both human and animal studies [48–50], suggesting that it could be a potential target for future antidepressant treatments. Further studies investigating these genes in acute phases would extend our understanding.

Lower levels of *OGG1* and *APE1* gene expression and higher levels of *POLβ* gene expression in siblings compared to HCs, indicate reduced DNA repair capacity in both patients with BD and their siblings. Many clinical, molecular, and neuroimaging studies have shown differences in first-degree relatives of individuals with BD compared to HCs [51]. Individuals who have a genetic predisposition to BD may display impairments in visual memory, verbal memory, processing speed, attention, and social cognition compared to HCs [52]. Structural and functional alterations in the prefrontal cortex [53–55] and white matter abnormalities in the body and splenium of the corpus callosum [56] have been observed in individuals with genetic predisposition to BD, as well as alterations in markers of neuroimmune dysregulation [57, 58]. Our results suggest that the BER pathway genes may serve as candidate endophenotypes for BD. In addition, dysregulated BER gene expressions may underlie the increased 8-OH-dG load in euthymic individuals with BD and their full siblings, potentially contributing to disease vulnerability and premature aging. However, further studies investigating BER genes and aging markers together are needed to confirm these associations.

The current study has several strengths. First, to the best of our knowledge, this is the first study to investigate the BER genes in the unaffected siblings of individuals with BD. Since full siblings of individuals with BD have the highest risk of developing the disorder after identical twins [59], the inclusion of full siblings of individuals with BD can provide valuable insights to demonstrate the DNA damage/repair abnormalities in individuals with a genetic predisposition of BD and can help identify potential targets for early intervention and prevention of BD. Additionally, mood-stabilizing medication is a major challenge in most studies on BD. The inclusion of full siblings who are genetically similar to individuals with BD but are not taking any medication helps to distinguish the potential impacts of medications. Second, using urine samples for 8-OH-dG measurement is another strength of the study, because it allows for the assessment of systemic oxidatively-induced DNA load in the body. Measurements of DNA damage markers in tissues can be affected by repair processes and may not fully reflect the oxidative load in the whole body. However, the products of oxidatively-induced DNA lesions, after repair, are excreted into urine without being further metabolized, making urinary 8-OH-dG levels a reliable indicator of systemic oxidatively-induced DNA damage [60, 61]. Additionally, the use of a gold standard technique for urine DNA damage measurements, liquid chromatography-tandem mass spectroscopy, for 8-OH-dG quantification is a strength due to its sensitivity and specificity compared to other techniques such as immunosorbent methods. To address a potential limitation resulting from the impact of glomerular filtration rate in measuring 8-OH-dG levels in urine samples [60], we used a normalization method by adjusting the 8-OH-dG levels to the levels of creatinine, as recommended by previous research [17]. Third, we have limited our cohort to individuals under the age of 50, as current literature defines old age as 50 in the BD population [62], in order to exclude the impact of senescence on our findings. Numerous studies have shown that age is a major confounding factor in research on oxidatively-induced DNA damage [63, 64]. Fourth, we controlled our findings for various types of confounders, including lifestyle factors, in addition to common confounders such as age, sex, BMI, smoking, and alcohol consumption. More importantly, investigating DNA damage and repair processes in combination allows us to gain a more comprehensive understanding of this dynamic process.

### Limitations

The five main limitations of this study must be considered and discussed. First, the cross-sectional nature of the study and the lack of longitudinal follow-up of patients and siblings limit the ability to draw causal conclusions and understand the direction of causality. Therefore, future follow-up of these siblings would be helpful in differentiating the roles of possible factors, such as resilience to BD, on these parameters. Second, some of the individuals recruited in the sibling group had a history of or currently presented psychiatric disorders. Although psychotic spectrum disorders and current depressive episodes were exclusion criteria due to their potential to cause a significant increase in 8-OH-dG levels, other psychiatric disorders such as past major depression, anxiety disorders, and attention deficit hyperactivity disorder were not considered as exclusion criteria in the sibling group. Although there are findings in the literature indicating increased DNA damage in these disorders [2, 46, 65], there is currently no available data on the extent to which this damage changes during the remission period. Additionally, our results showed that the current comorbid psychiatric illness in the sibling group did not affect DNA damage and BER mechanisms in the regression analysis. However, the potential influence of past or present psychiatric disorders on DNA damage and repair mechanisms should be considered in future studies with larger sample sizes. Third, another limitation of our study is that we did not exclude smokers, despite the well-established influence of smoking on these markers. However, due to the high prevalence of smoking in patients with BD and individuals with a genetic predisposition to BD, excluding smokers would introduce potential sampling bias. On the other hand, to address this limitation, we controlled for the effect of smoking in all statistical analyses. Fourth, our study focused only on the levels of 8-OH-dG lesion and four key BER genes involved in the repair of 8-OH-dG damage. Further studies investigating other types of DNA lesions, as well as RNA lesions, and the full pathway of BER genes will provide more comprehensive knowledge. Additionally, only focusing on mRNA expressions of genes is a limitation because protein levels and enzymatic activity may also affect the BER. Future studies using more comprehensive methods to investigate the BER enzymes may provide a more comprehensive understanding of the entire process. Finally, it is important to note that our study investigated DNA damage/repair status in peripheral blood cells, while brain cells have a high metabolic rate with high oxygen turnover, they may have a greater reliance on efficient and active BER than peripheral blood cells. On the other hand, studies suggest that there are consistent results among peripheral and central levels of DNA damage markers [66, 67]. Nevertheless, future studies investigating the DNA damage/repair status in brain tissue may provide a more accurate reflection of the mechanisms involved in BD.

## Conclusion

Our findings suggest that abnormalities in DNA damage and repair mechanisms are linked to familial susceptibility to BD. Given that increased levels of 8-OH-dG have also been observed in other psychiatric and somatic diseases, elevated levels of 8-OH-dG may indicate a shared mechanism of increased oxidatively-induced DNA damage and cellular aging, which may contribute to the comorbidity risk in individuals with a genetic predisposition to BD. On the other hand, the BER pathway abnormalities might be a potential underlying accumulation of DNA damage, leading to premature aging and an increased risk of comorbidities such as metabolic disorders, cardiovascular diseases, and neurodegenerative diseases in individuals with a genetic predisposition to BD. Hence, targeting the BER pathway might offer promising therapeutic strategies for reducing the risk of age-related diseases and comorbidities in individuals with mood disorders. Further, large-scale longitudinal studies investigating both oxidatively-induced DNA damage and BER pathways in BD are required to obtain more accurate and precise results.

## Data Availability

All data will be made available upon request.
