# Correction: Oxidatively-induced DNA base damage and base excision repair abnormalities in siblings of individuals with bipolar disorder DNA damage and repair in bipolar disorder

**DOI:** 10.1038/s41398-024-03051-2

**Published:** 2024-08-12

**Authors:** Hidayet Ece Arat Çelik, Selda Yılmaz, İzel Cemre Akşahin, Burcu Kök Kendirlioğlu, Esma Çörekli, Nazlı Ecem Dal Bekar, Ömer Faruk Çelik, Neşe Yorguner, Bilge Targıtay Öztürk, Hüray İşlekel, Ayşegül Özerdem, Pınar Akan, Deniz Ceylan, Gamze Tuna

**Affiliations:** 1 Department of Psychiatry, School of Medicine, Maltepe University, Istanbul, Turkey; 2 Department of Molecular Medicine, Institute of Health Sciences, Dokuz Eylul University, Izmir, Turkey; 3 Graduate School of Health Sciences, Koc University, Istanbul, Turkey; 4 Research Center for Translational Medicine (KUTTAM), School of Medicine, Koc University, Istanbul, Turkey; 5 Chair of Proteomics and Bioanalytics, School of Life Sciences, Technical University of Munich, Munich, Germany; 6 Department of Medical Biochemistry, Sancaktepe Sehit Prof. Dr. Ilhan Varank Training and Research Hospital, Istanbul, Turkey; 7 Department of Psychiatry, School of Medicine, Marmara University, Istanbul, Turkey; 8 Department of Psychiatry, School of Medicine, Dokuz Eylul University, Izmir, Turkey; 9 Department of Medical Biochemistry, School of Medicine, Dokuz Eylul University, Izmir, Turkey; 10 Department of Psychiatry and Psychology, Mayo Clinic, Rochester, MN USA; 11 Department of Neuroscience, Institute of Health Sciences, Dokuz Eylul University, Izmir, Turkey; 12 BioIzmir - Izmir Health Technologies Development and Accelerator Research and Application Center, Dokuz Eylul University, Izmir, Turkey; 13 Department of Psychiatry, School of Medicine, Koc University, Istanbul, Turkey

**Keywords:** Molecular neuroscience, Diagnostic markers

Correction to: *Translational Psychiatry* 10.1038/s41398-024-02901-3, published online 24 May 2024

In this article the funding from ‘[2021.KB.SAG.047]’ was omitted.

The corrected acknowledgment should read:

This research was funded by the Dokuz Eylul University Scientific Research Project Scholarship (2021.KB.SAG.047) and LithiumAssociation Scholarship.

The original article has been corrected.

